# USP35 promotes HCC development by stabilizing ABHD17C and activating the PI3K/AKT signaling pathway

**DOI:** 10.1038/s41420-023-01714-5

**Published:** 2023-11-22

**Authors:** Linpei Wang, Jiawei Wang, Xiaoqiu Ma, Guomin Ju, Chunfeng Shi, Wei Wang, Jian Wu

**Affiliations:** 1 Department of Hepatobiliary and Pancreatic Surgery, The Second Affiliated Hospital of Fujian Medical University, 362000 Quanzhou, Fujian Province China; 2 Division of Hepatobiliary and Pancreatic Surgery, Department of Surgery, The First Affiliated Hospital, Zhejiang University School of Medicine, 310003 Hangzhou, Zhejiang Province China; 3 Department of Health Medicine, The 910th Hospital of People’s Liberation Army, 362000 Quanzhou, Fujian Province China

**Keywords:** Hepatocellular carcinoma, Ubiquitylation

## Abstract

S-palmitoylation is a reversible protein lipidation that controls the subcellular localization and function of targeted proteins, including oncogenes such as N-RAS. The depalmitoylation enzyme family ABHD17s can remove the S-palmitoylation from N-RAS to facilitate cancer development. We previously showed that ABHD17C has oncogenic roles in hepatocellular carcinoma (HCC) cells, and its mRNA stability is controlled by miR-145-5p. However, it is still unclear whether ABHD17C is regulated at the post-translational level. In the present study, we identified multiple ubiquitin-specific proteases (USPs) that can stabilize ABHD17C by inhibiting the ubiquitin-proteasome-mediated degradation. Among them, USP35 is the most potent stabilizer of ABHD17C. We found a positive correlation between the elevated expression levels of USP35 and ABHD17C, together with their association with increased PI3K/AKT pathway activity in HCCs. USP35 knockdown caused decreased ABHD17C protein level, impaired PI3K/AKT pathway, reduced proliferation, cell cycle arrest, increased apoptosis, and mitigated migration and invasion. USP35 can interact with and stabilize ABHD17C by inhibiting its ubiquitination. Overexpression of ABHD17C can rescue the defects caused by USP35 knockdown in HCC cells. In support of these in vitro observations, xenograft assay data also showed that USP35 deficiency repressed HCC development in vivo, characterized by reduced proliferation and disrupted PI3K/AKT signaling. Together, these findings demonstrate that USP35 may promote HCC development by stabilization of ABHD17C and activation of the PI3K/AKT pathway.

## Introduction

Hepatocellular carcinoma (HCC) is the most common type of liver cancer, with over 750,000 cases reported annually [1–3]. Several risk factors have been identified to be associated with HCC, including cirrhosis, severe liver fibrosis, alcohol abuse, hepatitis B/C virus infection, and metabolic disorders [4–6]. Although novel drugs like sorafenib have been used to treat intermediate and advanced HCC, the treatment of HCC remains challenging and inefficient due to drug resistance, and side effects [7, 8].

S-palmitoylation is a reversible process in which the 16-carbon saturated fatty acid is covalently linked to Cys residues of targeted protein [9]. This modification plays an indispensable role in regulating the distribution of proteins in the cytosol and membrane [10–12], thereby influencing various biological and pathological processes [13, 14]. ABHD17C is a member of the newly discovered ABHD17 depalmitoylase family, which removes S-palmitoylation from the substrate [15, 16]. We previously reported that miR-145-5p regulates ABHD17C mRNA stability in HCC cells [17], but the post-translational modifications of ABHD17C remain unclear.

The ubiquitin-proteasome system is the major protein degradation system responsible for more than 80% of degradation events [18]. Ubiquitination, mediated by ubiquitin ligases, can be erased by deubiquitinases (DUBs). About 100 DUBs have been discovered in human cells [19, 20]. The ubiquitin-specific protease (USP) family is the largest family of DUBs in humans [19, 21]. While 54 human USPs share a conserved S1 ubiquitin-binding domain, their substrate interacting domain is diverse and not conserved, resulting in a significant variation in substrate binding capacity and specificity [21, 22]. Furthermore, the expression patterns and levels of different USPs contribute to their distinct functions in various contexts [23]. In HCC, several USPs have been reported to regulate HCC development [24–30], but the role of USP35 in HCC remains elusive.

In this study, we identified USP35 as a potent ABHD17C stabilizer in HCC cells. USP35 and ABHD17C were both upregulated in human HCC tissues and correlated with the PI3K/AKT pathway. Knockdown of USP35 resulted in accelerated degradation of ABHD17C, which in turn attenuated the PI3K/AKT pathway, reduced cell proliferation, induced cell cycle arrest, increased apoptosis, and mitigated migration and invasion of HCC cells. USP35 interacts with ABHD17C and deubiquitinates it, thereby increasing its stability and extending its half-life in HCC cells. Furthermore, overexpression of ABHD17C could rescue the defects in HCC cells caused by USP35 deficiency. We also observed that USP35 knockdown repressed the development of xenograft HCC tumors in vivo. In summary, these results suggest that USP35 may promote HCC development by stabilizing ABHD17C, thereby facilitating tumor progress through the activation of the PI3K/AKT signal cascade.

## Results

### USP35 stabilizes ABHD17C in HEK293T cells

**Fig. 1:**
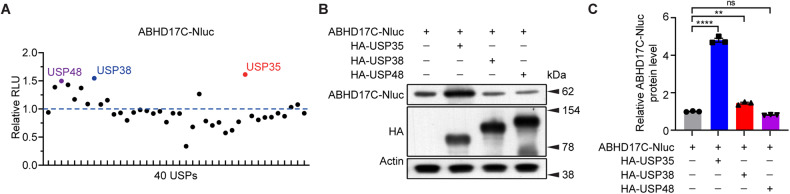
Multiple USPs regulate the stability of ABHD17C. **A** Normalized luciferase activity of 293T cells transfected with ABHD17C-Nluc and individual USPs. Cells expressing USP35, USP38, or USP48 exhibited the highest luciferase activity. **B** Immunoblot results confirmed the overexpression of USP35, USP38, and USP48 in HEK293T cells, and that the expression of ABHD17C was the highest in the USP35 overexpressing cells. **C** The quantification results in **B**. RLU relative light units.

To determine whether the stability of ABHD17C is regulated by USPs, HEK293T cells were co-transfected with luciferase-conjugated ABHD17C (ABHD17C-Nluc) and individual USPs overexpression plasmids. We found that multiple USPs significantly enhanced luciferase activity, indicating that these USPs increase the stability of ABHD17C (Fig. 1A). The top three most potent stabilizers, namely USP35, USP38, and USP48 were selected for immunoblot assays, which confirmed that USP35 displayed the highest potency in stabilizing ABHD17C in HEK293T cells (Fig. 1B, C). These results provided evidence that the stability of ABHD17C was regulated by the ubiquitin system, particularly by USP35.

### The expression levels of USP35 and ABHD17C are upregulated in human HCCs

**Fig. 2:**
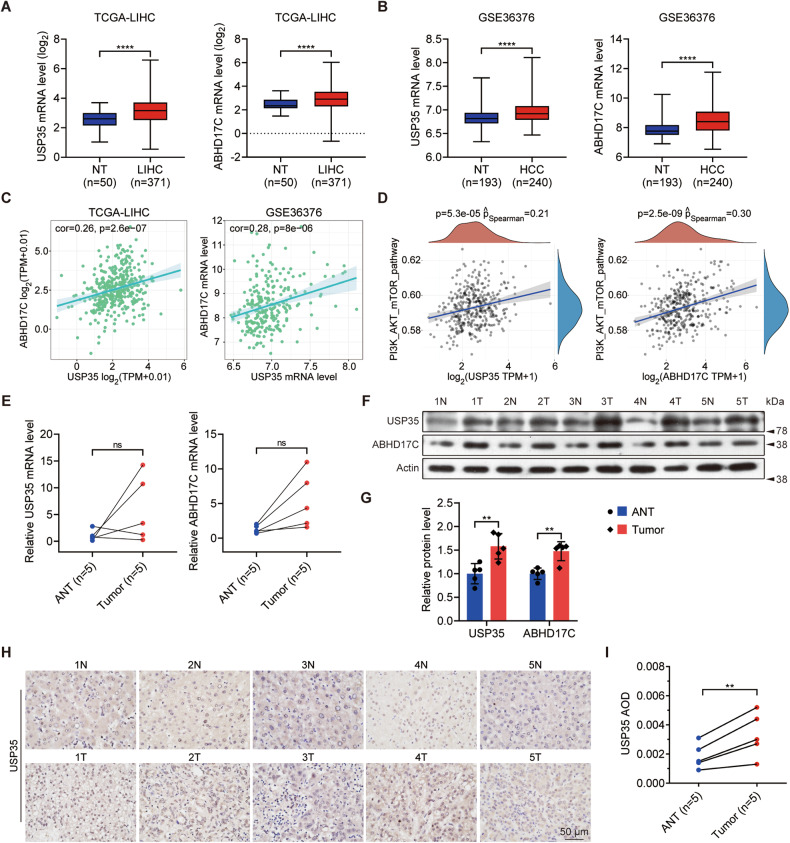
The expression levels of USP35 and ABHD17C are increased in human HCC samples and positively correlated with HCC and activated PI3K/AKT pathway. **A** Bioinformatic analysis results indicated that the mRNA levels of USP35 and ABHD17C were upregulated in the HCC tissues compared to non-tumorous (NT) tissues in the TCGA-LIHC database cohort. **B** The mRNA levels of USP35 and ABHD17C were also upregulated in the HCC tissues compared to non-tumorous (NT) tissues in the GSE36376 cohort. **C** Correlation analysis results showed a significant correlation between the expression of USP35 and ABHD17C in HCC samples from both cohorts. **D** GSEA analysis data indicated a correlation between USP35 or ABHD17C expression and the PI3K/AKT pathway. **E** qPCR results uncovered no significant change in the levels of USP35 and ABHD17C mRNA in HCC tumorous tissues compared to adjacent non-tumorous (ANT) tissues. **F** Immunoblot data indicated higher protein levels of USP35 and ABHD17C in HCC tumorous tissues compared to ANT tissues. **G** The quantification results of the data in **F**. **H**, **I** IHC data showed elevated expression of USP35 in HCC tumorous samples compared to ANT. ns not significant.

We then asked whether ABHD17C and USP35 mRNA levels were changed in human HCC samples. We downloaded RNA-seq data from public databases and performed bioinformatic analysis. The results from two independent datasets revealed that both USP35 and ABHD17C levels are upregulated in HCCs compared to control samples (Fig. 2A, B). Furthermore, a significant correlation was observed between the expression levels of ABHD17C and USP35 (Fig. 2C). As activation of the PI3K/AKT pathway is known to promote HCC development [31–33], we performed Gene Set Enrichment Analysis (GSEA) analysis and discovered a significant correlation between the expression level of USP35 or ABHD17C and the activation of PI3K/AKT pathway (Fig. 2D). To determine whether USP35 and ABHD17C are differentially expressed in tumorous and adjacent non-tumorous tissue (ANT) of HCC, we collected 5 HCC samples and evaluated their expression by qPCR and immunoblot. We didn’t observe a significant change in USP35 or ABHD17C mRNA levels but found a remarkable increase in USP35 and ABHD17C protein levels in tumorous tissues compared with that of ANT (Fig. 2E–G). Consistently, IHC results confirmed that USP35 expression in HCCs is higher than that in ANT (Fig. 2H, I). Taken together, these observations indicate that the increased expression of USP35 and ABHD17C in HCC may help activate the PI3K/AKT pathway.

### Knockdown of USP35 causes reduced proliferation, cell cycle arrest, increased apoptosis, and mitigated migration and invasion of HCC cells

**Fig. 3:**
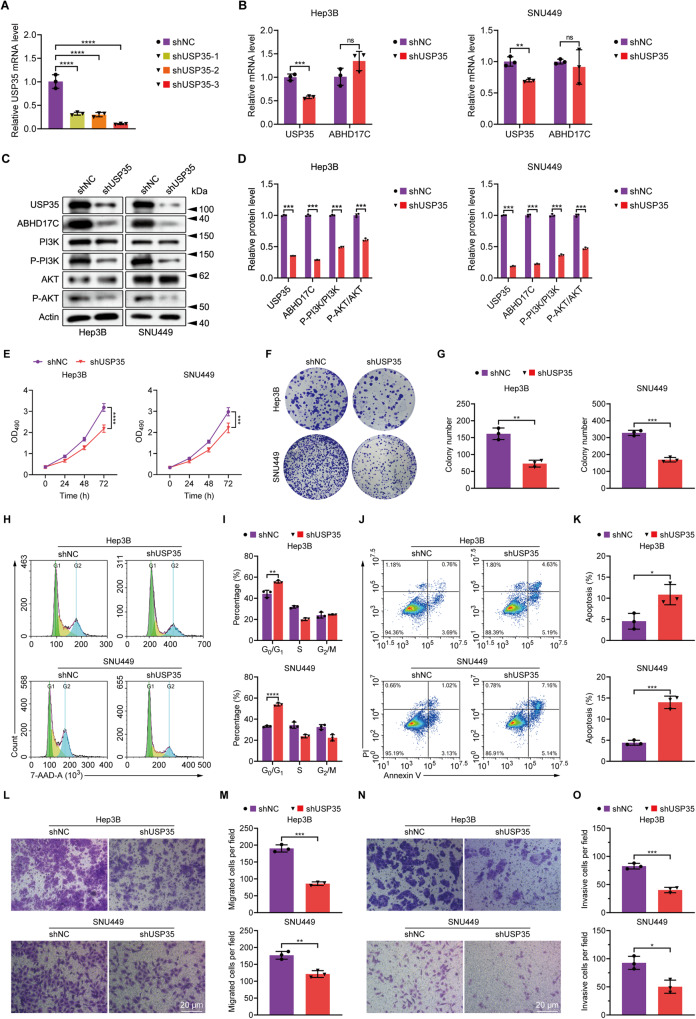
USP35 knockdown causes decreased expression of ABHD17C protein, impaired PI3K/AKT pathway, reduced proliferation, cell cycle arrest, increased apoptosis, and attenuated migration and invasion of HCC cells. **A** qPCR results demonstrated that shRNAs targeting USP35 efficiently reduce the mRNA levels of USP35. **B** The qPCR data revealed that shUSP35 significantly reduces the mRNA levels of USP35 in Hep3B and SNU449 HCC cells while having minimal effect on ABHD17C mRNA level. **C** Immunoblot data show that USP35, ABHD17C, p-PI3K, and p-AKT were down-regulated in USP35-deficient HCC cells compared to control HCC cells. **D** The quantification results of the data in **C**. **E** MTT assay results suggested that the proliferation of HCC cells was reduced by USP35 knockdown. **F** Colony formation assay data demonstrated that USP35-deficient HCC cells form fewer colonies compared to control cells. **G** The quantification results of the data in **F**. **H** 7-AAD staining results indicated that USP35 knockdown leads to more HCC cells being arrested in the *G*_0_/*G*_1_ phase. **I** The quantification results of the data in **H**. **J** Annexin-V/PI staining data revealed that USP35 knockdown triggers additional apoptosis in HCC cells. **K** The quantification results of the data in **J**. **L** Transwell assay data showed that USP35 knockdown mitigated the migration of HCC cells. **M** The quantification results of the data in **L**. **N** Transwell assay data indicated that USP35 knockdown impaired the invasion of HCC cells. **O** The quantification results of the data in **N**.

To investigate the function of USP35 in HCC, we first evaluated its expression in multiple HCC cell lines and observed that the USP35 mRNA level was remarkably elevated in multiple cell lines compared to the non-HCC LO-2 cells (Fig. S1A). Interestingly, the ABHD17C mRNA level was also elevated in the HCC cell lines which expressed high levels of USP35 mRNA (Fig. S1B). Consistent with this, USP35 and ABHD17C protein levels were also upregulated in most of these HCC lines (Fig. S1C–E). We then interfered with USP35 expression in Hep3B and SNU449 cells as they exhibited higher levels of USP35. We designed three shRNAs to target USP35 and found that all of them can efficiently knockdown USP35 expression in HEK239T cells (Fig. 3A). The shUSP35-3, which exhibited the highest knockdown efficiency, was selected for all the subsequent USP35 knockdown assays in Hep3B and SNU449 cells. We found that USP35 mRNA level was significantly reduced by shUSP35-3 in both HCC lines, while the mRNA levels of ABHD17C were unaffected (Fig. 3B). In line with these data, we observed that USP35 protein levels were reduced in Hep3B and SNU449 cells by shUSP35 (Fig. 3C, D). In contrast to unchanged mRNA levels, the protein levels of ABHD17C were greatly decreased in USP35-deficient HCC cells, and phosphorylated PI3K (p-PI3K) and AKT (p-AKT), which indicates the activation of PI3K/AKT pathway, were also significantly repressed in USP35-deficient HCC cells compared to control cells (Fig. 3C, D). Functional assays indicated that USP35 knockdown in Hep3B and SNU449 cells resulted in reduced cell proliferation, as evidenced by MTT and colony formation assays (Fig. 3E–G). Additionally, USP35 knockdown led to an increased number of cells arrested in the G_0_/G_1_ phase (Fig. 3H, I), as well as enhanced apoptosis in HCC cells (Fig. 3J, K). Moreover, USP35 knockdown impaired the migration and invasion capabilities of HCC cells (Fig. 3L–O). Collectively, these observations demonstrated the oncogenic functions of USP35 in HCC cells, which is consistent with the function of ABHD17C.

### USP35 interacts with and stabilizes ABHD17C in HCC cells

**Fig. 4:**
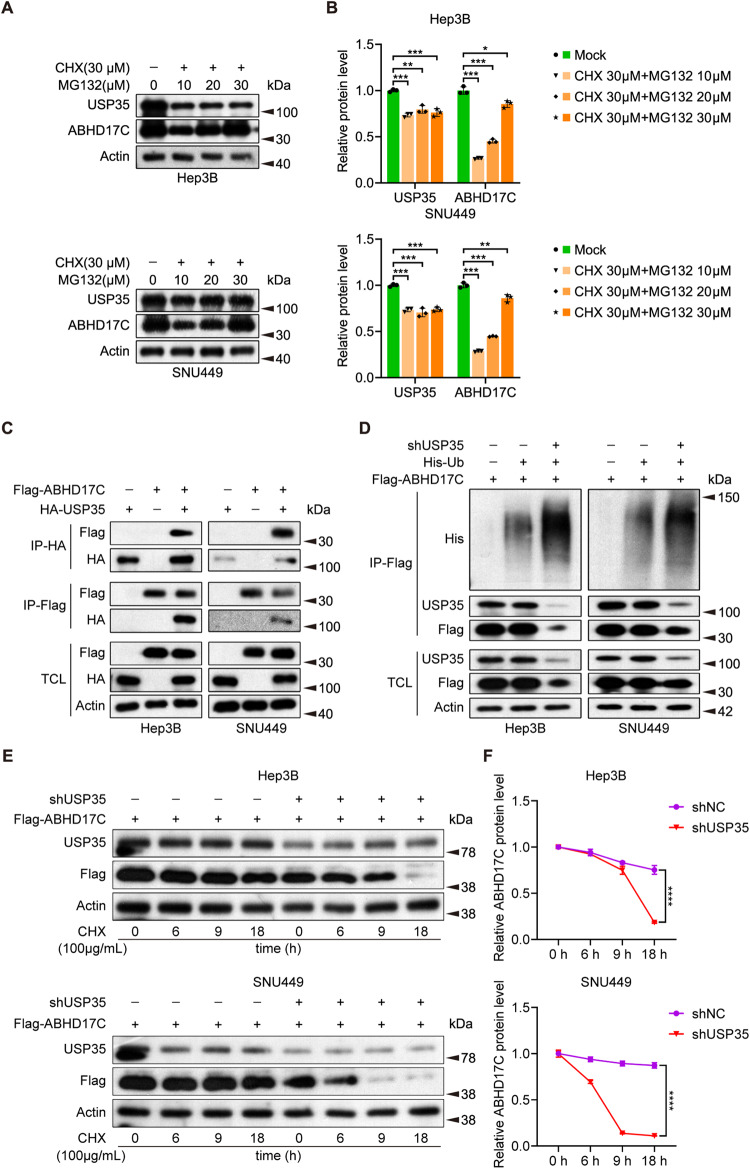
USP35 interacts with and deubiquitinates ABHD17C in HCC cells. **A** Immunoblot data indicated that MG132 treatment can dose-dependently stabilize ABHD17C in HCC cells. **B** The quantification results of the data in **A**. **C** Co-IP results revealed the interaction between USP35 and ABHD17C in Hep3B and SNU449 cells. **D** Immunoblot results displayed a significant increase in the ubiquitination of ABHD17C in USP35-deficient HCC cells compared to control cells. **E** Immunoblot data revealed that USP35 knockdown accelerated the degradation of ABHD17C in HCC cells. **F** The quantification results of the data in **E**. TCL total cell lysate.

Since USP35 stabilizes ABHD17C in HEK293T cells, we tested whether USP35 controls the stability of ABHD17C in HCC cells. We treated the Hep3B and SNU449 cells with cycloheximide (CHX) to prevent protein synthesis and added different doses of MG132, a proteasome inhibitor. We discovered that MG132 treatment dose-dependently inhibited the degradation of ABHD17C in the presence of CHX in both HCC cell lines (Fig. 4A, B), suggesting that the stability of ABHD17C is also modulated by the ubiquitin system in HCC cells. To find out whether USP35 directly interacts with ABHD17C in HCC cells, we overexpressed HA-USP35 and Flag-ABHD17C in Hep3B and SNU449 cells and performed co-IP assays. Co-IP results showed that USP35 and ABHD17C interact with each other (Fig. 4C). To determine whether the ubiquitination of ABHD17C is controlled by USP35, we introduced His-tag labeled Ubiquitin (His-ub) and pulled down the ABHD17 from control and USP35-deficient HCC cells. We discovered that the ubiquitination of ABHD17C was greatly elevated by USP35 knockdown (Fig. 4D). In support of this, we found that USP35 deficiency significantly accelerated ABHD17C degradation in HCC cells (Fig. 4E, F). Collectively, these findings demonstrated that USP35 interacted with and stabilized ABHD17C by regulating its ubiquitination in HCC cells.

### Overexpression of ABHD17C rescues the defects caused by USP35 knockdown in HCC cells

**Fig. 5:**
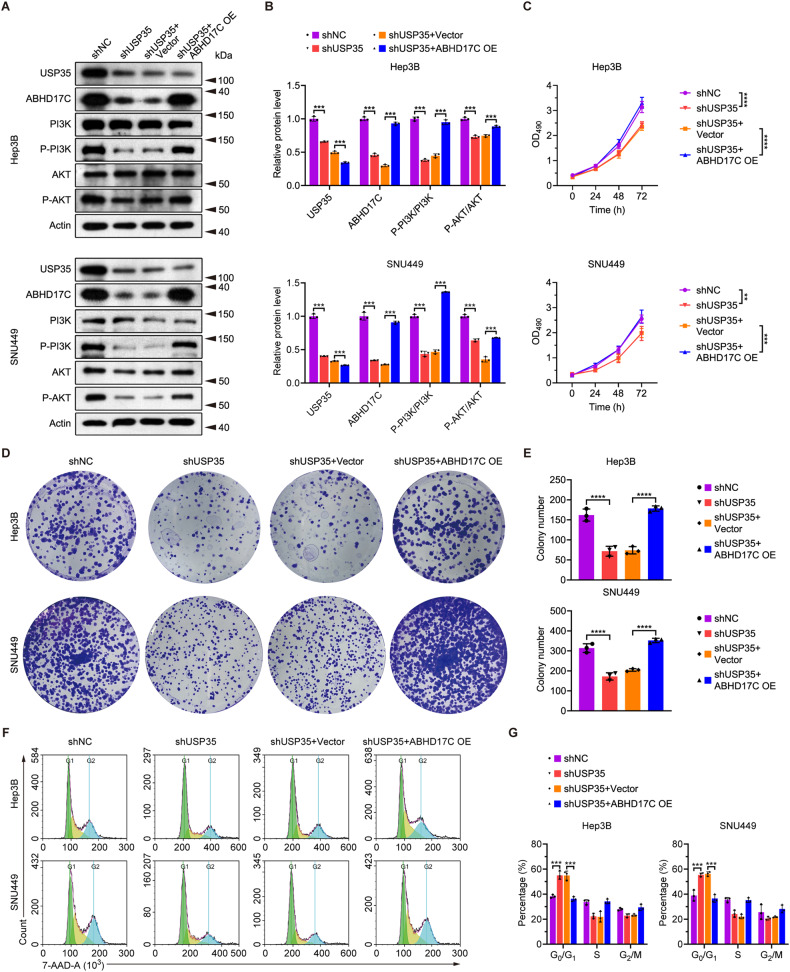
Overexpression of ABHD17C rescues the proliferation defects of USP35-deficient HCC cells. **A** Immunoblot data demonstrated that the overexpression of ABHD17C in USP35-deficient Hep3B and SNU449 cells restored the levels of p-PI3K and p-AKT to levels comparable to those in control cells. **B** The quantification results of the data in **A**. **C** MTT assay results revealed that the proliferation of USP35-deficient HCC cells was rescued by excessive ABHD17C. **D** Colony formation assay data showed that the colony formation ability of USP35-deficient HCC cells was rescued by overexpression of ABHD17C. **E** The quantification results of the data in **D**. **F** 7-AAD staining assay demonstrated that the cell cycle arrest of HCC cells caused by USP35 deficiency was rescued by overexpression of ABHD17C. **G** The quantification results of the data in **F**. OE overexpression.

**Fig. 6:**
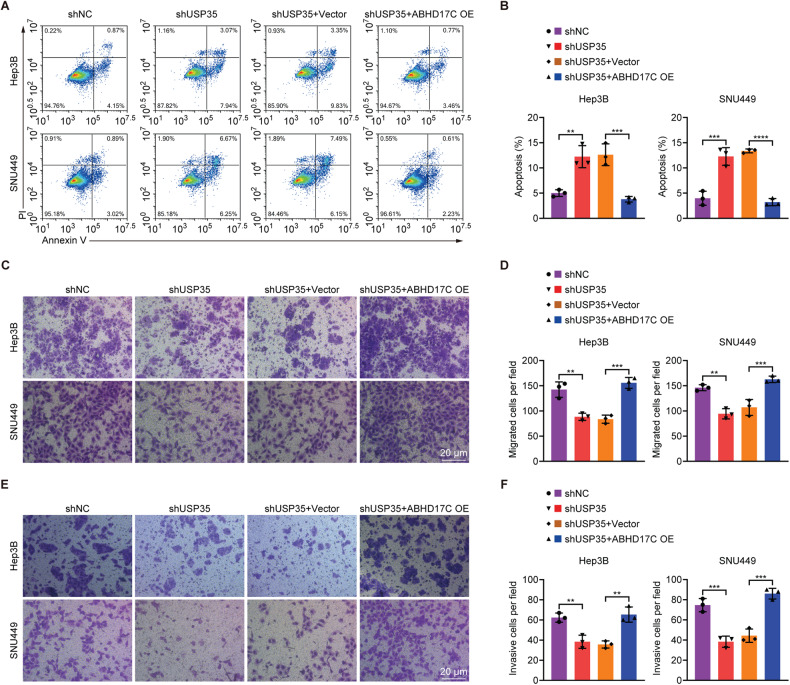
Overexpression of ABHD17C rescues the apoptosis, migration, and invasion defects of USP35-deficient HCC cells. **A** The increased apoptosis of USP35-deficient HCC cells was alleviated by excessive ABHD17C, as evidenced by the Annexin-V/PI staining assay. **B** The quantification results of the data in **A**. **C** Transwell assay results showed that the mitigated migration of USP35-deficient HCC cells was rescued by overexpression of ABHD17C. **D** The quantification results of the data in **C**. **E** The impaired invasion of USP35-deficient HCC cells was rescued by overexpression of ABHD17C, as evidenced by Transwell assay results. **F** The quantification results of the data in **E**. OE overexpression.

To decide whether the oncogenic function of USP35 in HCC cells relies on ABHD17C, we overexpressed ABHD17C in USP35-deficient Hep3B and SNU449 cells and found that ABHD17C overexpression rescued the reduced levels of p-PI3K and p-AKT caused by USP35 knockdown (Fig. 5A, B). In addition, the decreased proliferation, cell cycle arrest, increased apoptosis, and impaired migration and invasion observed in USP35-deficient HCC cells were all restored to levels comparable to those of control cells upon excessive ABHD17C expression (Figs. 5C–G, and 6A–F). These data indicated that ABHD17C played an important part in mediating the oncogenic function of USP35 in HCC cells.

### USP35 deficiency suppresses HCC development in vivo

**Fig. 7:**
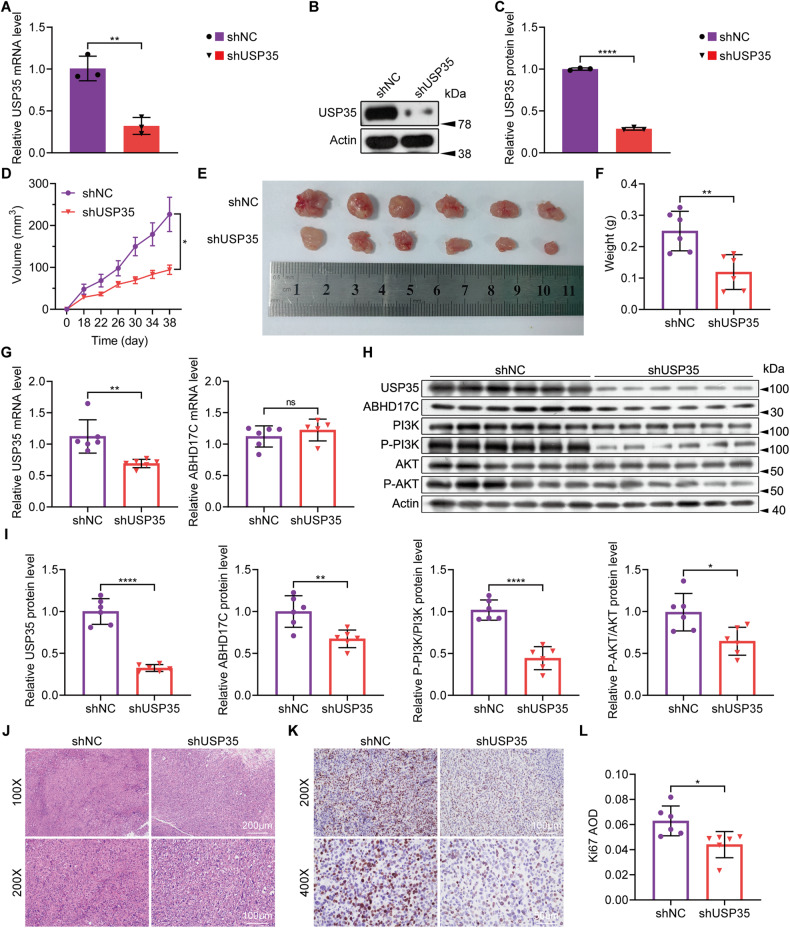
USP35 knockdown suppresses HCC xenograft tumor development in vivo. **A** qPCR results confirmed efficient knockdown of USP35 mRNA expression in stable Hep3B cells transfected with shUSP35. **B** Immunoblot results showed a significant reduction in USP35 expression in the USP35-deficient stable Hep3B cells. **C** The quantification results of the data in **B**. **D** Tumor volume curve depicting the growth of xenograft tumors. **E** Morphology of xenograft tumors at the endpoint. **F** The quantification results of the weight of xenograft tumors at the ending point. **G** qPCR results demonstrated significantly lower mRNA levels of USP35 in xenograft tumors from USP35-deficient Hep3B cells compared to control tumors. However, the mRNA levels of ABHD17C were comparable between the control and USP35-deficient xenograft tumors. **H** Immunoblot data showed significantly reduced protein levels of USP35, ABHD17C, p-PI3K, and p-AKT in USP35-deficient xenograft tumors compared to control tumors. **I** The quantification results of the data in **H**. **J** H&E staining results indicated lower tumor cell density in USP35-deficient xenograft tumors compared to control tumors. **K**, **L** IHC data for Ki-67 showed decreased proliferation of USP35-deficient xenograft tumor cells compared to control xenograft tumor cells.

To explore whether USP35 regulates HCC development in vivo, we established stable USP35-deficient Hep3B cells through lentivirus infection and screening (Fig. 7A–C). We then injected control and USP35-deficient Hep3B cells into nude mice to generate xenograft HCC models. We observed that the size and weight of USP35-deficient tumors were significantly reduced compared to the control tumors (Fig. 7D–F). Consistent with previous in vitro results, the ABHD17C mRNA levels were not affected by USP35 knockdown in the xenograft tumors (Fig. 7G). However, the protein levels of ABHD17C, p-PI3K, and p-AKT were greatly down-regulated in USP35-deficient tumors (Fig. 7H, I). Moreover, the density and proliferation of xenograft tumor cells were also reduced by USP35 knockdown as indicated by H&E staining and Ki-67 IHC results (Fig. 7J–L). Together, these data suggest that USP35 promotes HCC development in vivo.

## Discussion

In this study, we explored the expression and function of USP35 and ABHD17C in HCC cells. We found that the expression of USP35 and ABHD17C are both elevated, and their expression levels are significantly correlated in HCC samples. Our data also demonstrate that USP35 promotes HCC development by stabilizing ABHD17C and activates the PI3K/AKT pathway.

The oncogenic function of USP35 in other cancers has been well characterized. For example, USP35 promotes ovarian cancer development by deubiquitinating and inactivating STING [34]. USP35 also facilitates the growth and progression of lung cancer cells by desensitizing them from ferroptosis through deubiquitination of ferroportin [35]. Moreover, the expression of USP35 is positively correlated with poor prognosis of non-small cell lung cancer (NSCLC) and represses endoplasmic reticulum stress-induced apoptosis by stabilizing RRBP1 [36]. In addition, USP35 can accelerate prostate cancer growth by deubiquitinating BRPF1 [37]. Our finding that USP35 positively regulates HCC development by interacting with and deubiquitinating ABHD17C further confirms the oncogenic roles of USP35 and identifies ABHD17C as its novel target in HCC cells. Given the diversity of its substrates, it will be important to investigate whether USP35 promotes HCC development by additional targets other than ABHD17C.

ABHD17 depalmitoylases can regulate palmitoylation of N-RAS to change its subcellular localization, and selective inhibitor of ABHD17 proteins is reported to repress N-RAS signaling and impair acute myeloid leukemia cell growth [15, 16]. Hyperactivation of N-RAS leads to activation of multiple oncogenic signaling pathways [38]. Among them, the PI3K/AKT signaling pathway is a master regulator of cancers [39]. In HCC, overactivation of RAS signaling transduction promotes hepatic cell transformation and hepatoma progression through MAPK and PI3K/AKT pathways [40–42]. Overexpression of N-RAS with AKT or in the Pten knockout mice can induce spontaneous HCC [43–45]. In line with these reports, we found that the PI3K/AKT pathway is disrupted in USP53-deficient HCC cells, which is likely caused by a lack of sufficient ABHD17C.

**Fig. 8:**
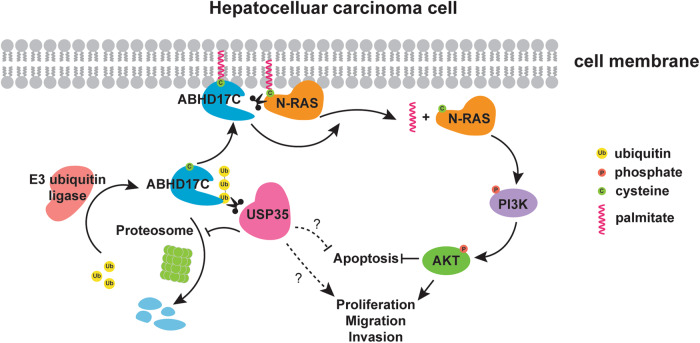
Schematic diagram of the function of USP35 in regulating HCC development. ABHD17C on the cell membrane removes palmitoylation of N-RAS to release N-RAS and facilitate N-RAS activation [16]. Activated N-RAS triggers the PI3K/AKT signal cascade to promote proliferation, migration, and invasion, but repress the apoptosis of HCC cells. ABHD17C is ubiquitinated by E3 ubiquitin ligase for proteosome-mediated degradation, which is reversed by USP35. As a result, ABHD17C is stabilized and continuously promotes N-RAS activation. Whether USP35 regulates HCC development by additional mechanisms (dash lines) needs further investigation.

In conclusion, our study provides evidence that USP35 promotes HCC development by stabilizing ABHD17C (Fig. 8). These findings expand our knowledge about HCC development and underscore the importance of USP35 and ABHD17C as potential therapeutic targets for HCC treatment.

## Materials and methods

### Cell lines

All cell lines assayed, including HEK293T, Hep3B, HepG2, Huh-7, MHCC-97H, PLC/PRF/5, SMMC-7721, SNU-182, SK-Hep-1, and LO-2 cells were obtained from Immocell Biotechnology (Xiamen, China). The cells were cultured in DMEM (Gibco, USA) with 10% FBS (Gibco, USA) and kept at 37 °C with 5% CO_2_ and appropriate humidity. Before being used for experiments, the cells were tested for Mycoplasma contamination using the Plasmo Test (InvivoGen, USA). Cycloheximide (CHX) and MG132 (proteasome inhibitor) obtained from MedChemExpress (USA) were subjected to the culture medium at the indicated concentrations. 3 biological replicates were used for phenotypic analysis.

### Patient samples and processing

Fresh and paraffin-embedded HCC tissues were obtained from The Second Affiliated Hospital of Fujian Medical University hospital with the permission of the hospital’s Ethics Committee (Approval number: No.27[2023]). Written informed consent was obtained from the patients, adhering to the principles of the Declaration of Helsinki. Paraffin-embedded tissues containing HCC, as well as adjacent non-tumorous tissue, were sectioned for IHC analysis. Fresh HCC tissues were used to extract RNA and protein, which were assayed for gene expression levels. 5 patient samples were included for analysis.

### Bioinformatic analysis

The RNA profiling and associated clinical data of HCC patients were acquired from TCGA-LIHC (https://portal.gdc.cancer.gov/projects/TCGA-LIHC) and GEO (GSE36376, https://www.ncbi.nlm.nih.gov/geoprofiles). These datasets were used to analyze the expression and pattern of *USP35* and *ABHD17C*, as well as their correlation. The relationship between *USP35* or *ABHD17C* and specific signaling pathways were analyzed by GSEA, with the R package “GSVA” and the method=’ssgsea’. p < 0.05 was considered significant.

### Plasmids

The plasmids pmirGLO-G4S-ABHD17C-Nluc and PRL-TK for luciferase reporter assay and Plasmids encoding Flag-tagged ABHD17C, HA-tagged USPs (USP1-8, USP9X, USP9Y, USP10-22, and USP24-40), His-tagged ubiquitin, or short hairpin RNA (shRNA) targeting USP35 were obtained from XIAMEN Anti-hela Biological Technology (Xiamen, China). Lentivirus for the construction of stable low-expression USP35 cell lines was purchased from XIAMEN Anti-hela Biological Technology (Xiamen, China).

### Cell transfection

Lipofectamine 3000 transfection reagent (Invitrogen, USA) was used for non-viral plasmid transfection. Briefly, plasmids were pre-incubated with Opti-MEM (Gibco, USA)-diluted Lipofectamine 3000 transfection reagent. The mixture was thoroughly mixed and then applied to cells of 70% confluence. After 48 h, the transfected cells were harvested for subsequence analyses, including luciferase activity measurement, and RNA or protein expression analysis.

### Construction of cell lines with stable low expression of USP35

For lentivirus infection, Hep3B and SNU449 cells were cultured until they reached 70% confluence and maintained in a fresh culture medium. Subsequently, viruses were diluted in Opti-MEM, and the dilution was added into cells based on the optimized multiplicity of infection (MOI). Sixteen hours later, the medium was replaced by fresh medium. Following a 48-h incubation period post-infection, the cells were incubated with a medium containing 3 μg/mL puromycin. After an additional 72-h culture, the surviving cells were harvested for further analyses of RNA and protein expression.

### Luciferase reporter assay

48 h after HA-USPs and pmirGLO-G4S-ABHD17C-Nluc transfection, the HEK293T cells were lysed using a passive lysis buffer. Subsequently, the luciferase activity was assayed with the Dual-Luciferase Reporter Assay System (Promega, USA) on the Varioskan LUX microplate reader (ThermoFisher Scientific, USA). Firefly luciferase activity was normalized to Renilla luciferase activity within each sample to calculate the level of ABHD17C protein.

### RNA extraction and quantitative PCR (qPCR)

RNA extraction and purification from cells or tumors were performed using Trizol reagent (Invitrogen, USA) according to the manufacturer’s protocol. Reverse transcription of RNA was carried out using HiScript Reverse Transcriptase (Vazyme, China). qPCR was performed on a LightCycler 96 instrument (Roche, USA) using ChamQ qPCR Mix (Vazyme, China). At least 3 biological replicates were performed in each group. RNA levels were calculated using the 2^−ΔΔCt^ method. The primers used for qPCR are listed in Table S1.

### Protein extraction, immunoprecipitation (IP), and immunoblot

Protein samples were prepared from cells or cancer tissues using the RIPA lysis buffer (Beyotime Biotechnology, China) according to the manufacturer’s protocol.

For IP assays, 2 μg antibody against Flag or HA tag (Proteintech, China) was incubated with Protein A + G magnetic beads (Beyotime Biotechnology, China) in Ab binding & washing buffer supplied in the immunoprecipitation kit (Invitrogen, USA) for 10 min. After that, the beads were washed and incubated with protein samples with gentle rotation and mixing for 2 h at room temperature. Then the supernatant was discarded, and the beads were washed three times. Finally, 20 μL elution buffer was applied to elute the precipitated protein samples for subsequent immunoblot analysis.

For immunoblot, samples were first mixed with loading buffer and denatured for 5 min at 95 °C. Proteins were then separated by electrophoresis and transferred to PVDF membranes, blocked and incubated with blocking solutions containing primary antibodies overnight at 4 °C. After rinsing, the membranes were immersed in blocking solution containing HRP-conjugated goat anti-rabbit IgG (#SA00001-2, Proteintech, China) for 2 h at room temperature. Finally, the membranes were rinsed with TBST three times before incubating with ECL reagent (Beyotime Biotechnology, China) for signal visualization. Protein levels were normalized to internal control using ImageJ (National Institutes of Health, USA). Three replication experiments were performed for quantification. The primary antibodies used are listed in Table S2. Full and uncropped western blot graphs are shown in Supplementary Material 2.

### MTT assay

MTT assay was performed using a detection kit (Beyotime Biotechnology, China) following the user’s guide. Briefly, cells were seeded into 96-well plates at a density of 3,000 cells per well. 10 μL MTT working solution (5 mg/mL) per well was added, and the plates were placed in a 37 °C incubator for 6 h. Subsequently, 100 μL/well DMSO was pipetted into the plates to resolve crystallization, and the plates were kept for another 3 h, followed by 10-min agitation at 37 °C on an oscillator at 300 rpm/min. Finally, the OD_490_ values were measured using a microplate reader. For quantification, three independent assays were carried out for each condition.

### Colony formation assay

Seven hundred cells per well were inoculated into six-well plates and cultured for 12 days. Subsequently, the media was discarded, and the cells were fixed with 4% paraformaldehyde for 30 min and stained with 0.5% crystal violet for 15 min. The colonies were then washed and photographed. ImageJ was used for colony number calculation. Three independent assays were carried out for each condition for quantification purposes.

### Cell cycle and apoptosis analysis

For cell cycle analysis, 5 × 10^6^ Hep3B or SNU449 cells were harvested and treated with ethanol overnight at −20 °C. Cells were then washed, resuspended by PBS, and treated with 20 μL RNase A for 30 min at 37 °C. After removal of RNAse A, the cells were resuspended with PBS containing 5 μL 7-AAD solution (Thermo Fisher Scientific, USA) and maintained in the dark at 4 °C for 30 min. Finally, the cells were subjected to flow cytometry analysis and the results were analyzed using Flowjo (BD Bioscience, USA).

For apoptosis detection assay, Annexin-V-FITC/PI apoptosis detection kit (Vazyme, China) was used following the manufacturer’s guide. Briefly, 5 × 10^5^ Hep3B or SNU449 cells were harvested, washed in PBS, and resuspended with 100 μL binding buffer in the kit. 5 μL Annexin-V-FITC and 5 μL PI solution were then mixed and administrated to the cells and incubated away from light at room temperature for 10 min. After that, 400 μL binding buffer was added and cells were analyzed by flow cytometry. All these experiments were performed in triplicate for quantification purposes.

### Transwell assay

Transwell assays were conducted using 24-well plates with 8 μm Transwell chambers (#3422, Corning, USA). For invasion analysis, 100 μL per chamber matrix glue was applied and the plates were kept at 37 °C for 2 h. The cells were then digested, washed, and resuspended with serum-free media at 3 × 10^5^ cells per mL. After that, 200 μL cell suspension was administrated to the top chamber and 700 μL complete medium was added to the bottom chamber. After 48-h culture, the cells were treated with methanol for 30 min at room temperature and then stained with 0.05% crystal violet (Solarbio, China) for 5 min. The results were photographed under an inverted microscope (IX73, OLYMPUS, Japan). ImageJ was used for image processing and data quantification. For migration analysis, the experimental procedures were identical to the invasion experiment except that matrix glue was not used. All the assays were repeated in triplicate for quantification purposes.

### Animals and cell-derived xenograft modeling

Male, 8-week-old BALB/c nu/nu mice (hereafter called nude mice) were obtained from Vital River Laboratory Animal Technology (Beijing, China). 2 × 10^6^ cells with or without USP35 deficiency were subcutaneously injected into each mouse and no randomization was used. Tumors were measured every other 4 days from 18 days post injection (dpi). All the mice were sacrificed at 38 dpi, and tumors were dissected, weighed, and photographed. After that the tumors were processed for subsequent assays including quantitative PCR (qPCR), immunoblot, and histology analysis. No blinding of investigators was involved during the experiments. All the experimental procedures strictly followed the guidelines of NIH and the guidelines of The Second Affiliated Hospital of Fujian Medical University, 6 individuals were included in each group.

### Histology and immunohistochemistry (IHC)

Xenograft tumors were fixed with 4% paraformaldehyde overnight at 4 °C and then processed for paraffin embedding. Paraffin sections of 4 μM thickness were then rehydrated and stained with the hematoxylin and eosin staining kit (Beyotime Biotechnology, China).

For IHC, sections were dewaxed and rehydrated. After antigen retrieval, the sections were treated with 3% hydrogen peroxide, permeabilized with 0.2% Triton X-100/PBS, and blocked with 1% BSA. Then, the sections were incubated with USP35 antibody (ab254939, Abcam, UK, 1:500) or Ki-67 antibody (ab15580, Abcam, UK, 1:500) at 4 °C overnight. After washing, the sections were incubated with HRP-conjugated goat anti-rabbit IgG (SA00001-2, Proteintech, China, 1:1000) at room temperature for 1 h. Subsequently, the sections were washed and stained with DAB working solution (Beyotime Biotechnology, China), counterstained with hematoxylin solution, and mounted. Finally, the sections were photographed by an IX73 inverted microscope (OLYMPUS, Japan). ImageJ was used for image processing and quantification of Ki-67+ cells.

### Statistical analysis

All comparisons between the two sets of quantitative data were performed using paired or unpaired two-tailed, two-sided Student *t*-tests, as the variances were similar between the groups under comparison. All comparisons among multiple sets of quantitative data were analyzed using one-way analysis of variance. Data were displayed as mean ± SD, and *p* < 0.05 was considered significant. *p* < 0.05, *p* < 0.01, *p* < 0.001, and *p* < 0.0001 were represented by *, **, *** and ****, respectively.

### Supplementary information

Supplymentary figure 1 and tables

Original Western Blot Data File

## Data Availability

The data that support the findings of this study are available on request from the corresponding authors, W Jian and WW, upon reasonable requests and reasons.
