# Supplementary material for: USP35 promotes HCC development by stabilizing ABHD17C and activating the PI3K/AKT signaling pathway
Source: Cell Death Discov. 2023 Nov 22;9:421. doi: 10.1038/s41420-023-01714-5 (PMC10665393; doi:10.1038/s41420-023-01714-5)
Supplement: Supplementary file 1 — Supplymentary figure 1 and tables [file 41420_2023_1714_MOESM1_ESM.docx]

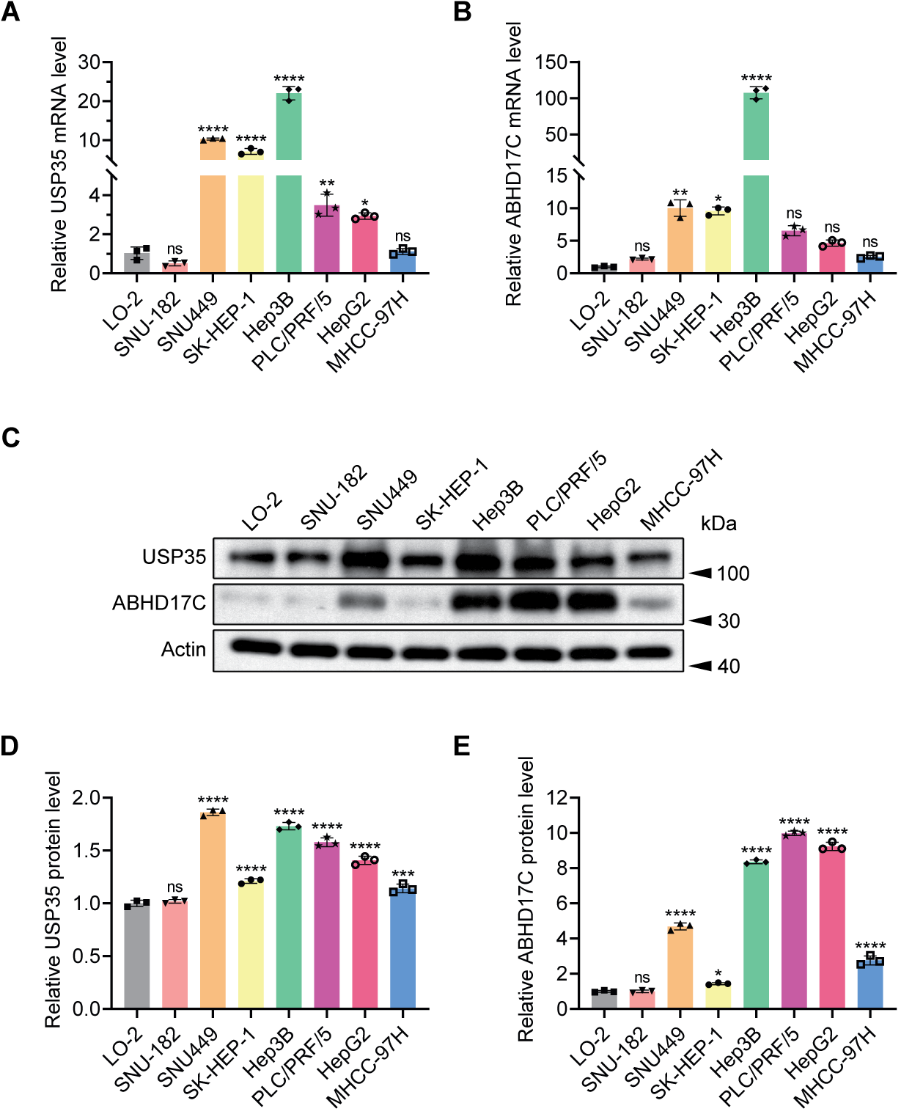


**Figure S1. The expression of USP35 and ABHD17C is elevated in multiple HCC cell lines.** (A) qPCR results showing USP35 mRNA levels in the indicated cell lines. (B) qPCR results showing ABHD17C mRNA levels in the indicated cell lines. (C) Immunoblot data showing USP35 and ABHD17C protein levels in the indicated cell lines. (D) The quantification results of the USP35 protein levels from immunoblot results. (E) The quantification results of the ABHD17C protein levels from immunoblot results.

**Table S1. The primers used for qPCR in this study**

| USP35-F | 5′-AGAGAACTTCCTCTCCGCATCC-3′ |
| --- | --- |
| USP35-R | 5′-CTGGACTGCTTGAGTTTCTGGC-3′ |
| ABHD17C-F | 5′-GCAGCGGTAATTCTCCATTCCC-3′ |
| ABHD17C-R | 5′-GACCAACACAGGAGAGGTGACT-3′ |
| RNA18SN5-F | 5′-ACCCGTTGAACCCCATTCGTGA-3′ |
| RNA18SN5-R | 5′- GCCTCACTAAACCATCCAATCGG-3′ |

**Table S2. Primary antibody applied in this study for immunoblot**

| Target protein | Manufacturer | Catalog number | Dilution |
| --- | --- | --- | --- |
| USP35 | Abcam, UK | ab254939 | 1:1000 |
| HA tag | Proteintech, China | 51064-2-AP | 1:5000 |
| ABHD17C | Invitrogen, USA | PA5-61831 | 1:1000 |
| β-actin | Proteintech, China | 20536-1-AP | 1:1000 |
| PI3K | Proteintech, China | 13329-1-AP | 1:1000 |
| Flag tag | Proteintech, China | 20543-1-AP | 1:20000 |
| AKT | Proteintech, China | 10176-2-AP | 1:2000 |
| Phospho-PI3K | Cell Signaling Technology, USA | 17366 | 1:1000 |
| Phospho-AKT | Cell Signaling Technology, USA | 9271 | 1:1000 |
