# Supplementary figures and images for: USP35 promotes HCC development by stabilizing ABHD17C and activating the PI3K/AKT signaling pathway
Source: Cell Death Discov. 2023 Nov 22;9:421. doi: 10.1038/s41420-023-01714-5 (PMC10665393; doi:10.1038/s41420-023-01714-5)

Figure 1B

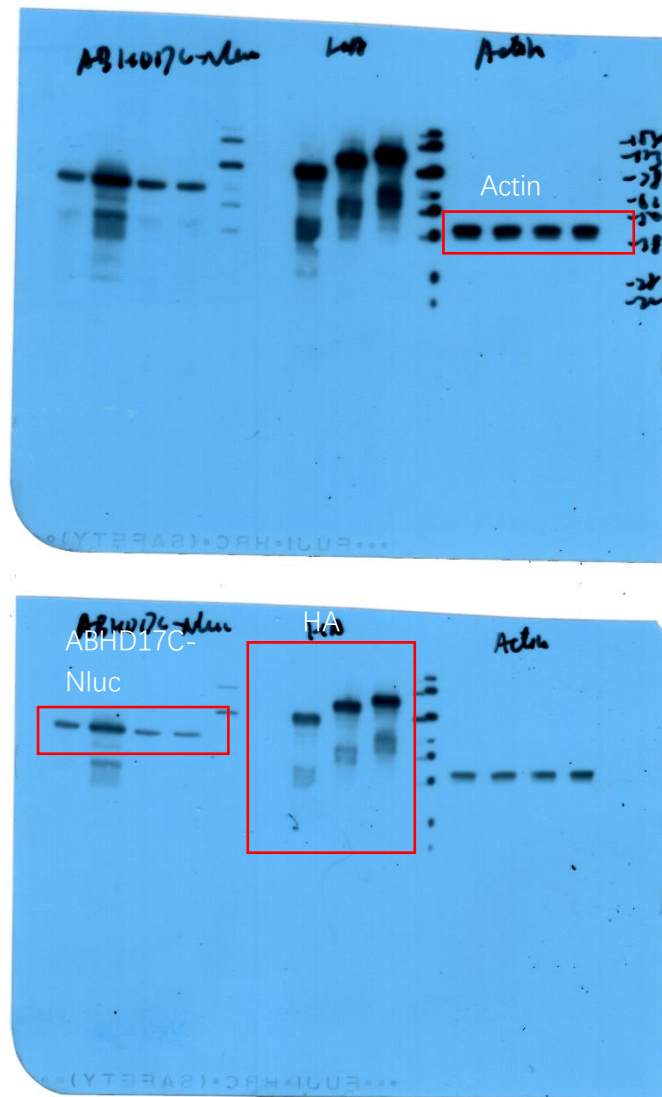

Figure 2F

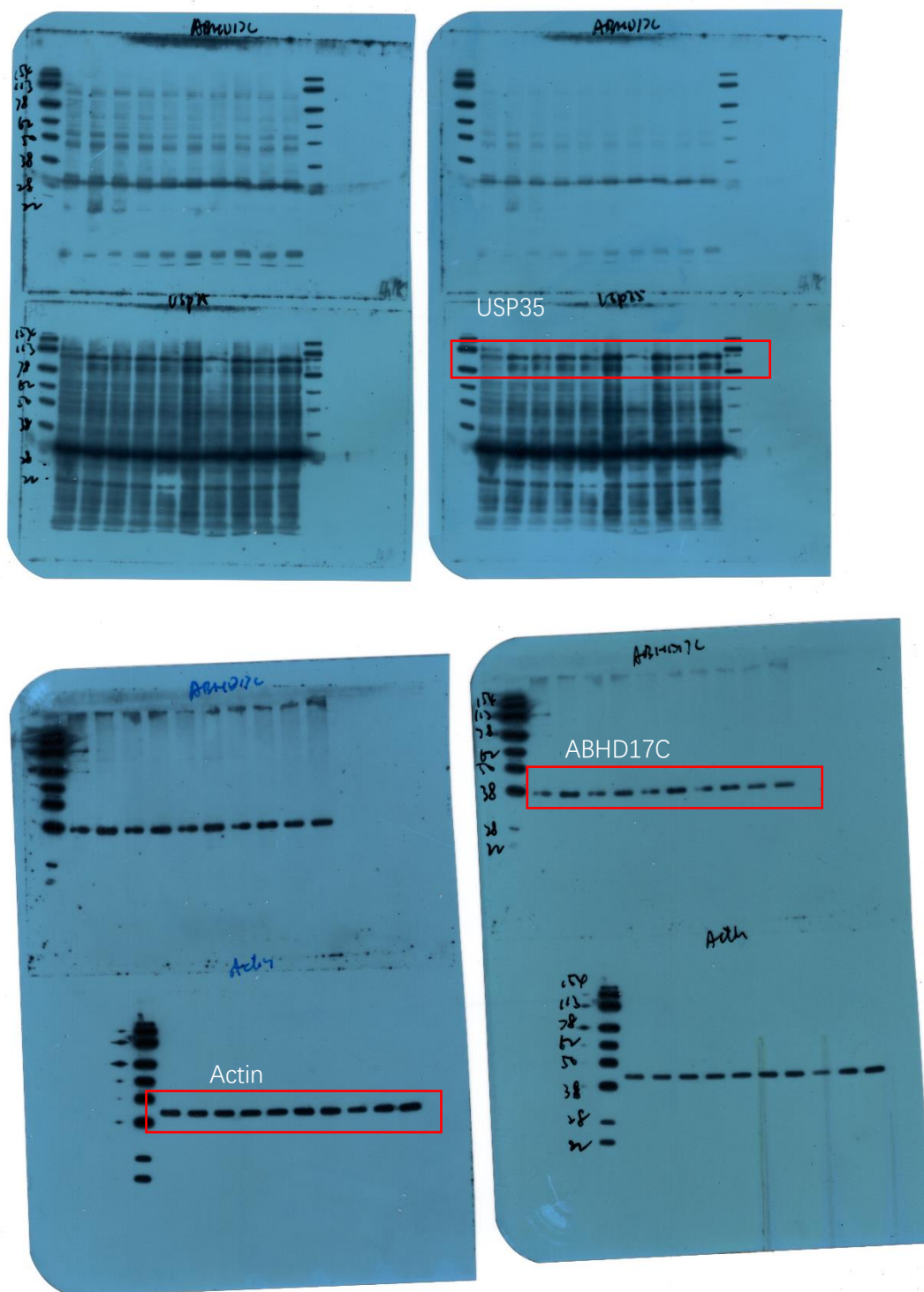

Figure 3C

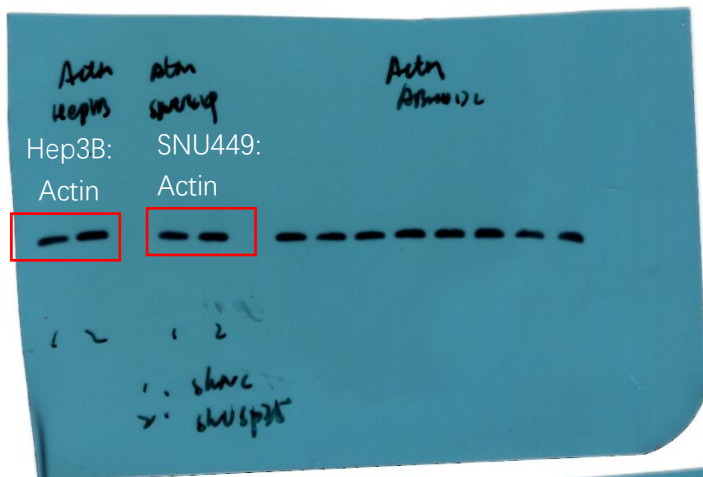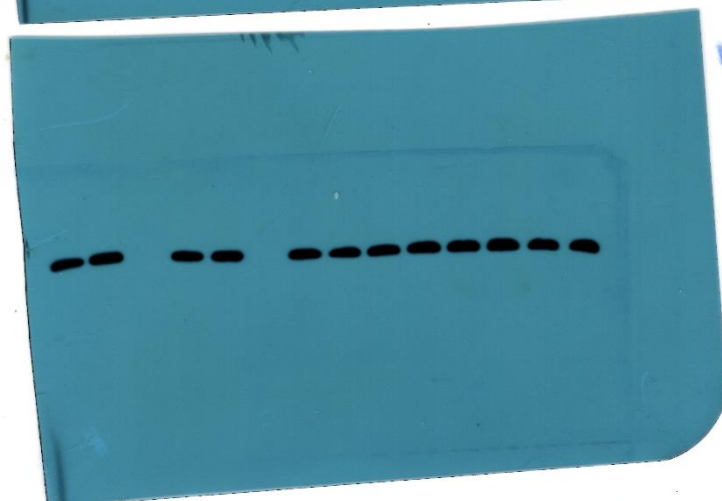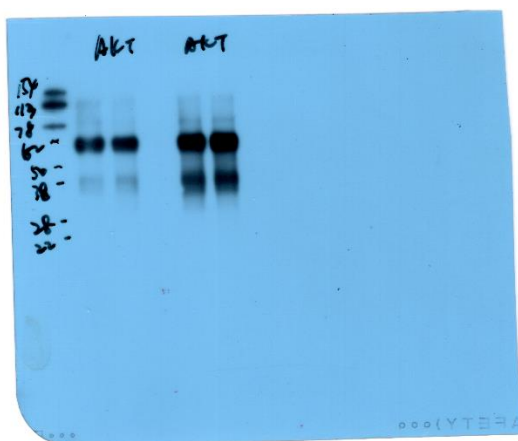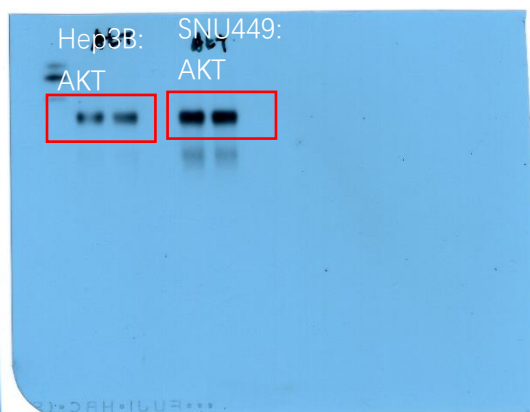

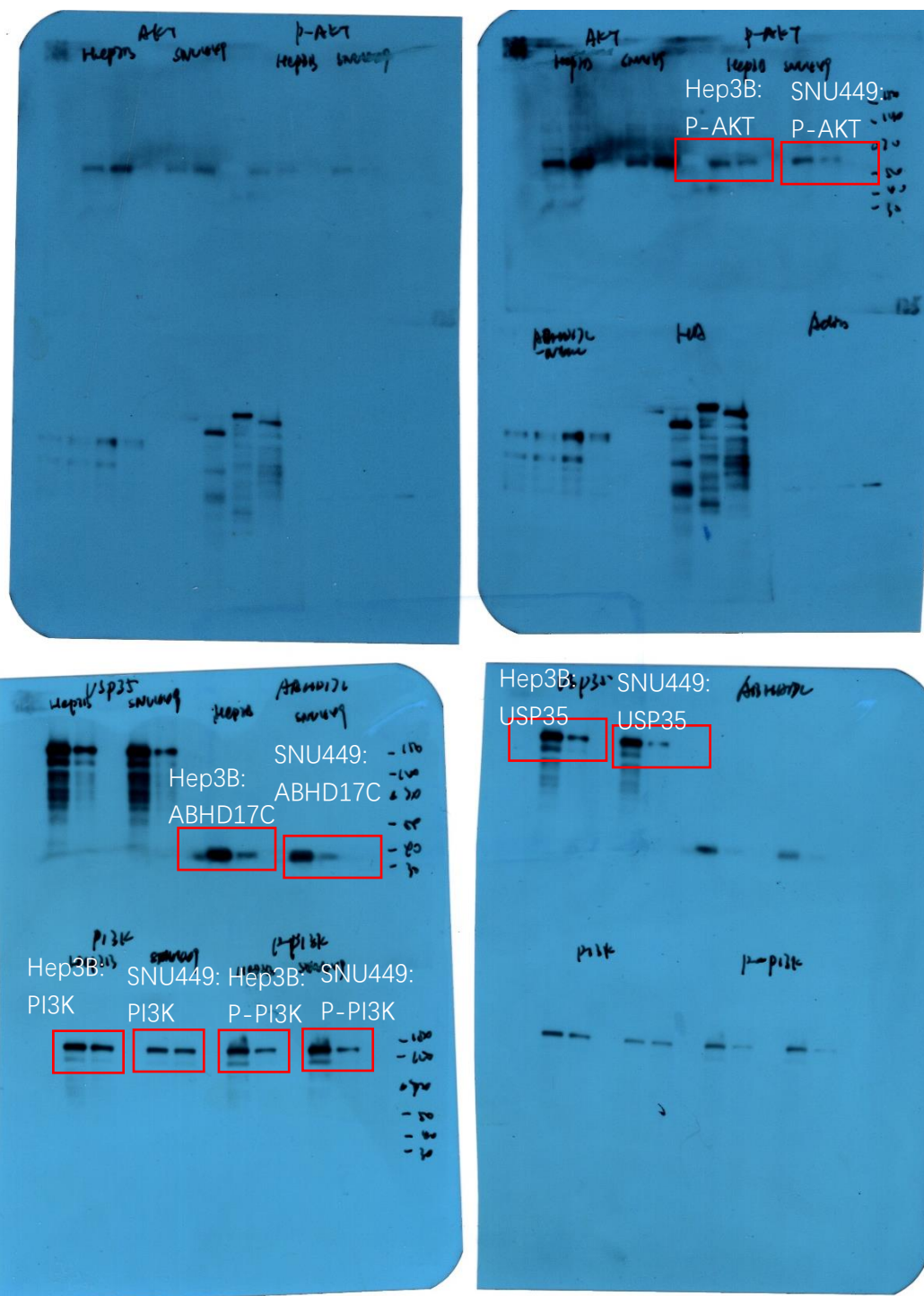

Figure 4A

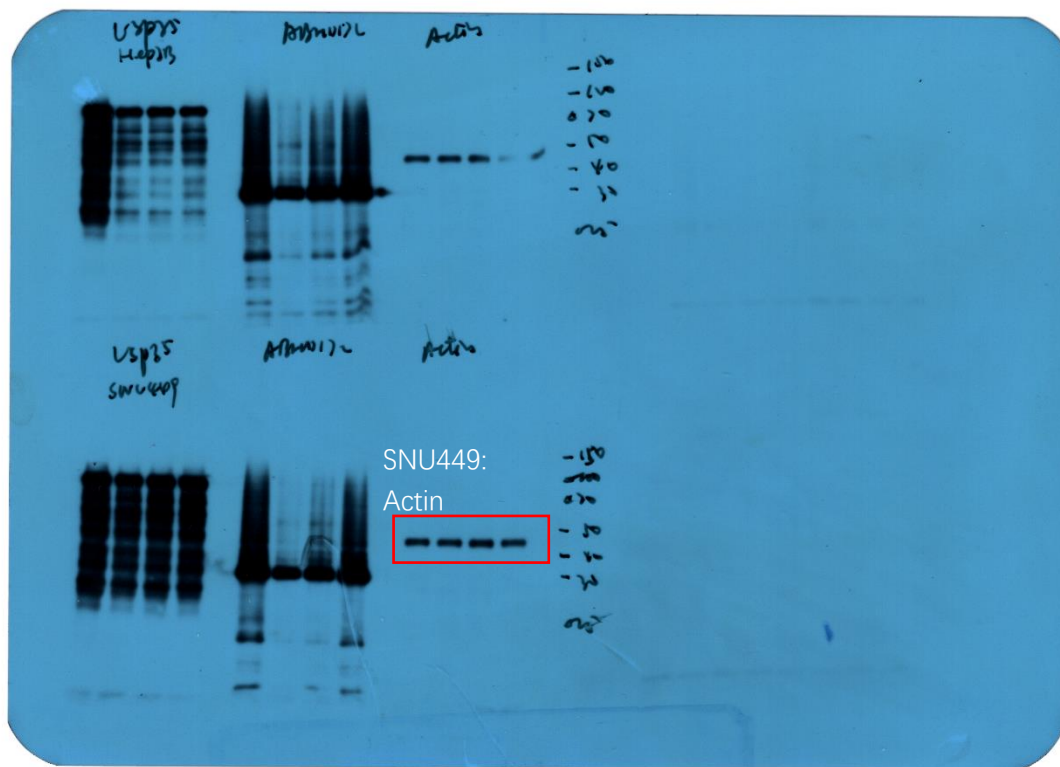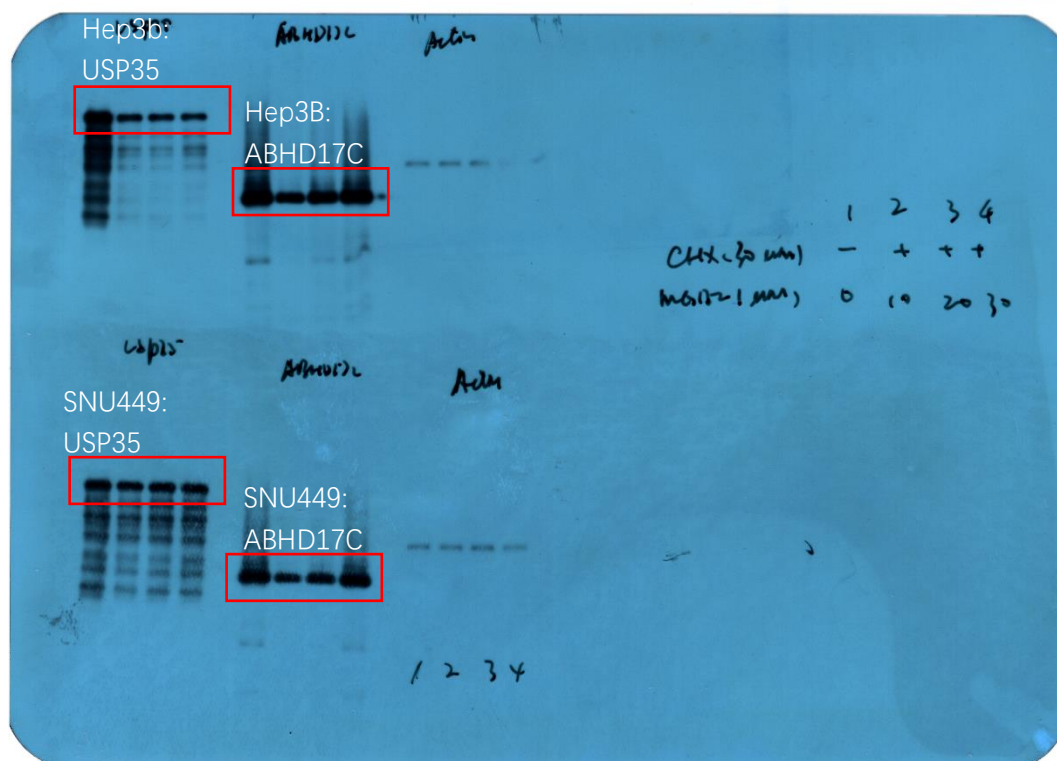

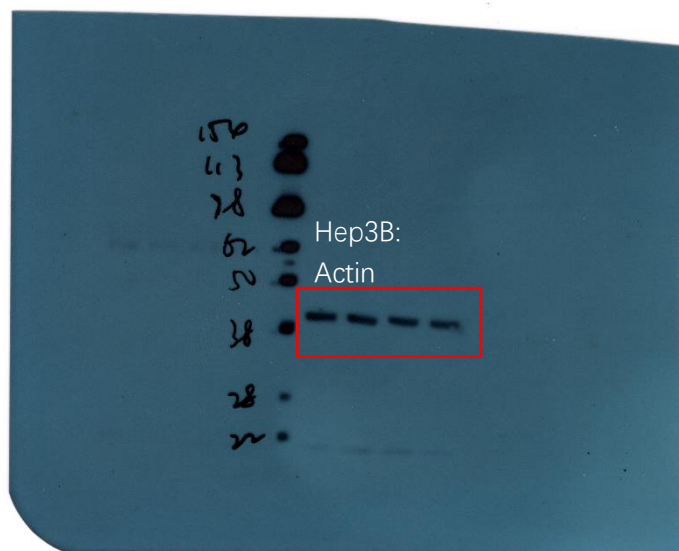

Figure 4C

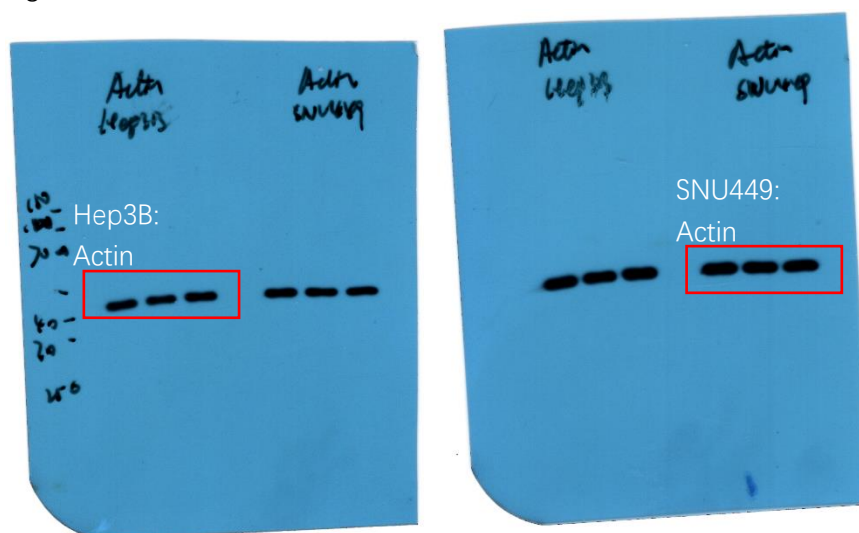



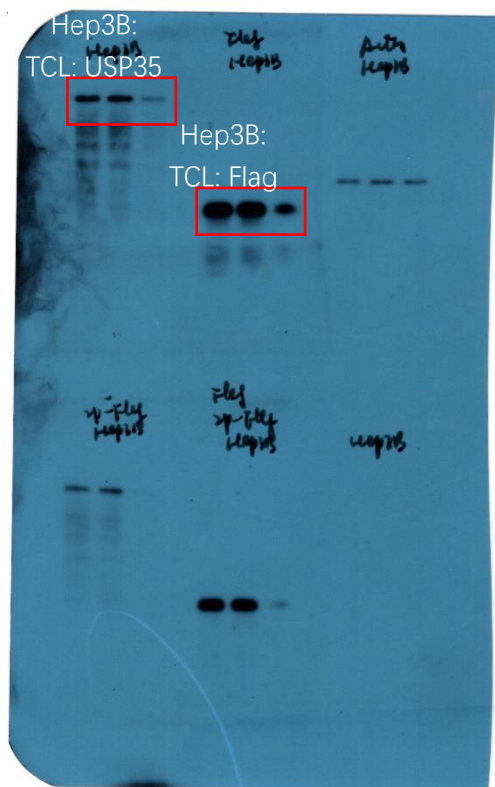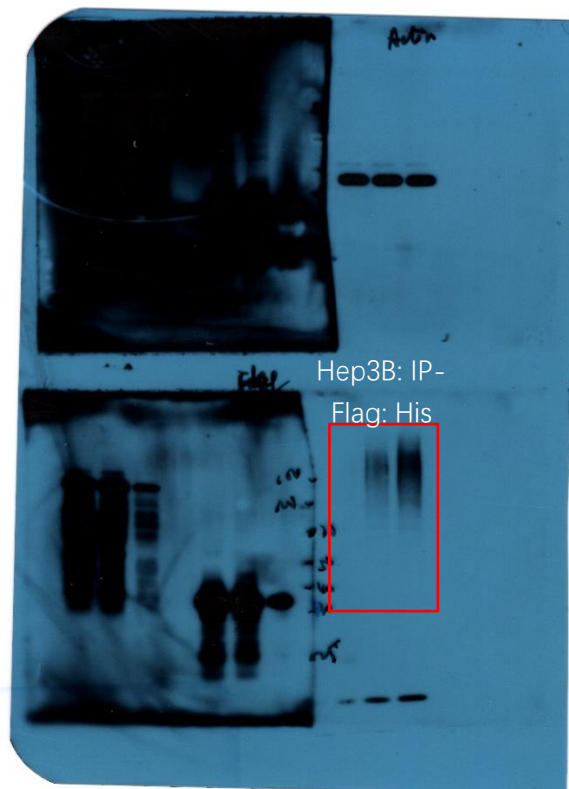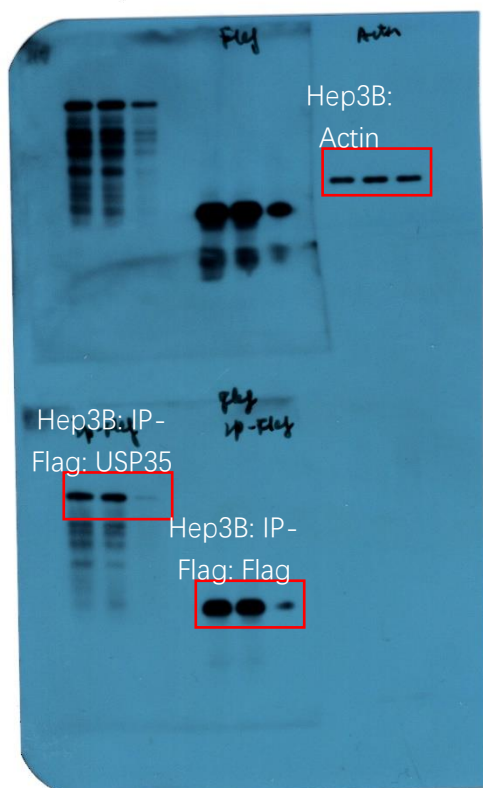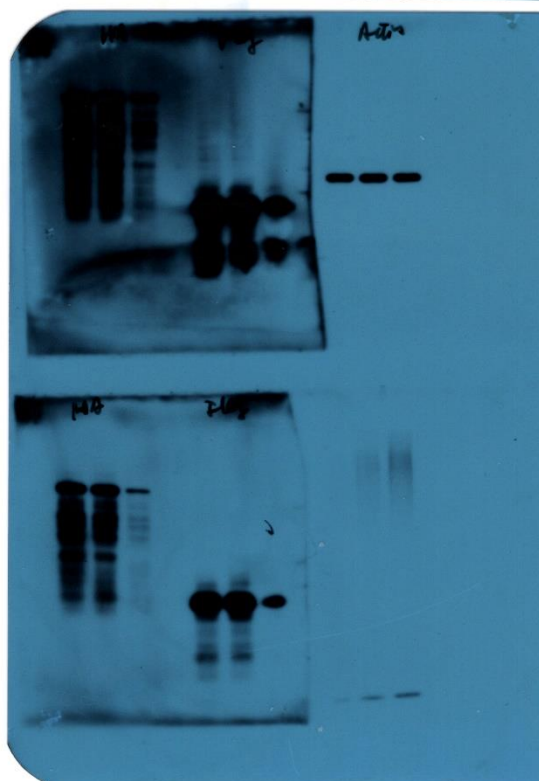

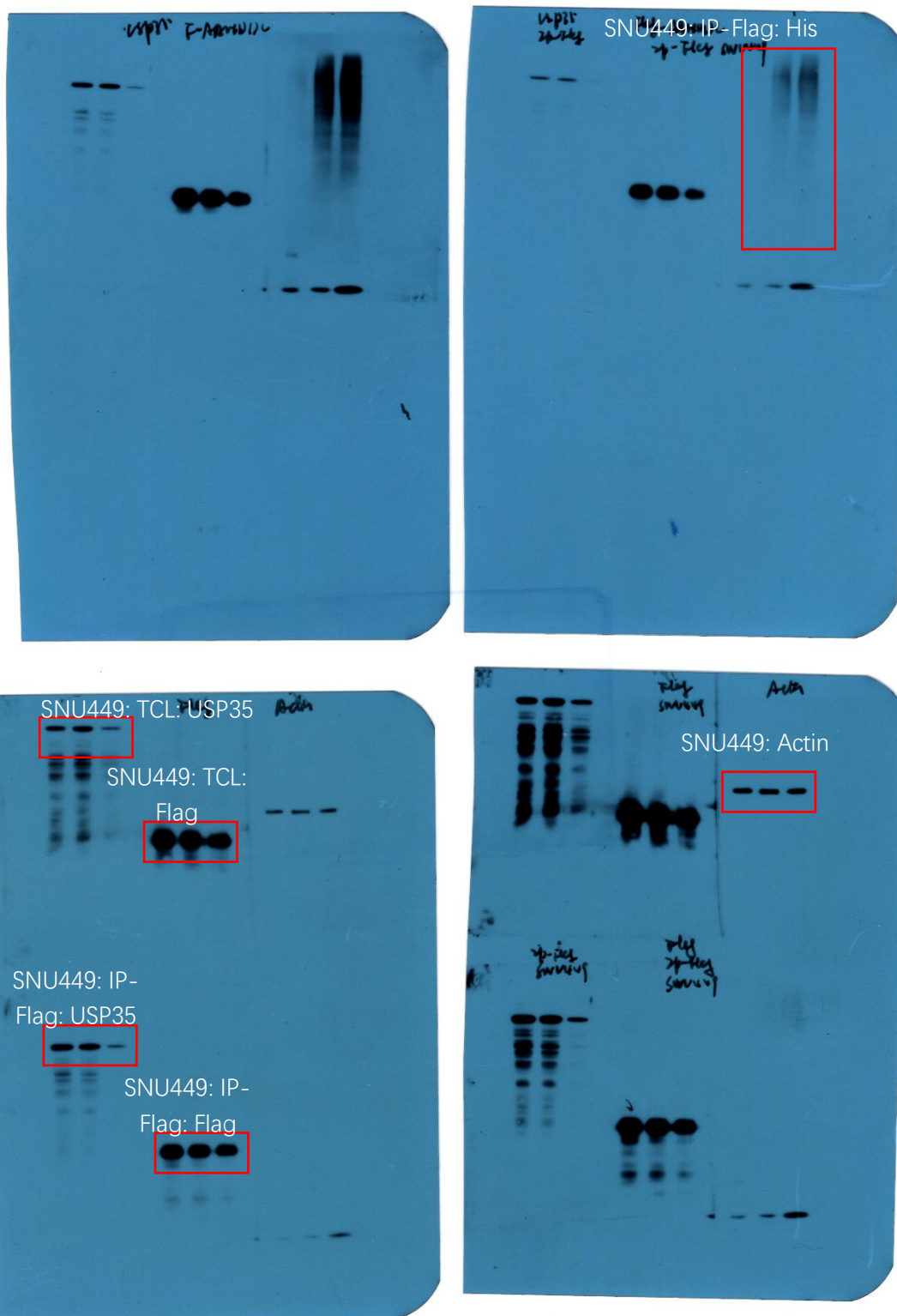

Figure 4E

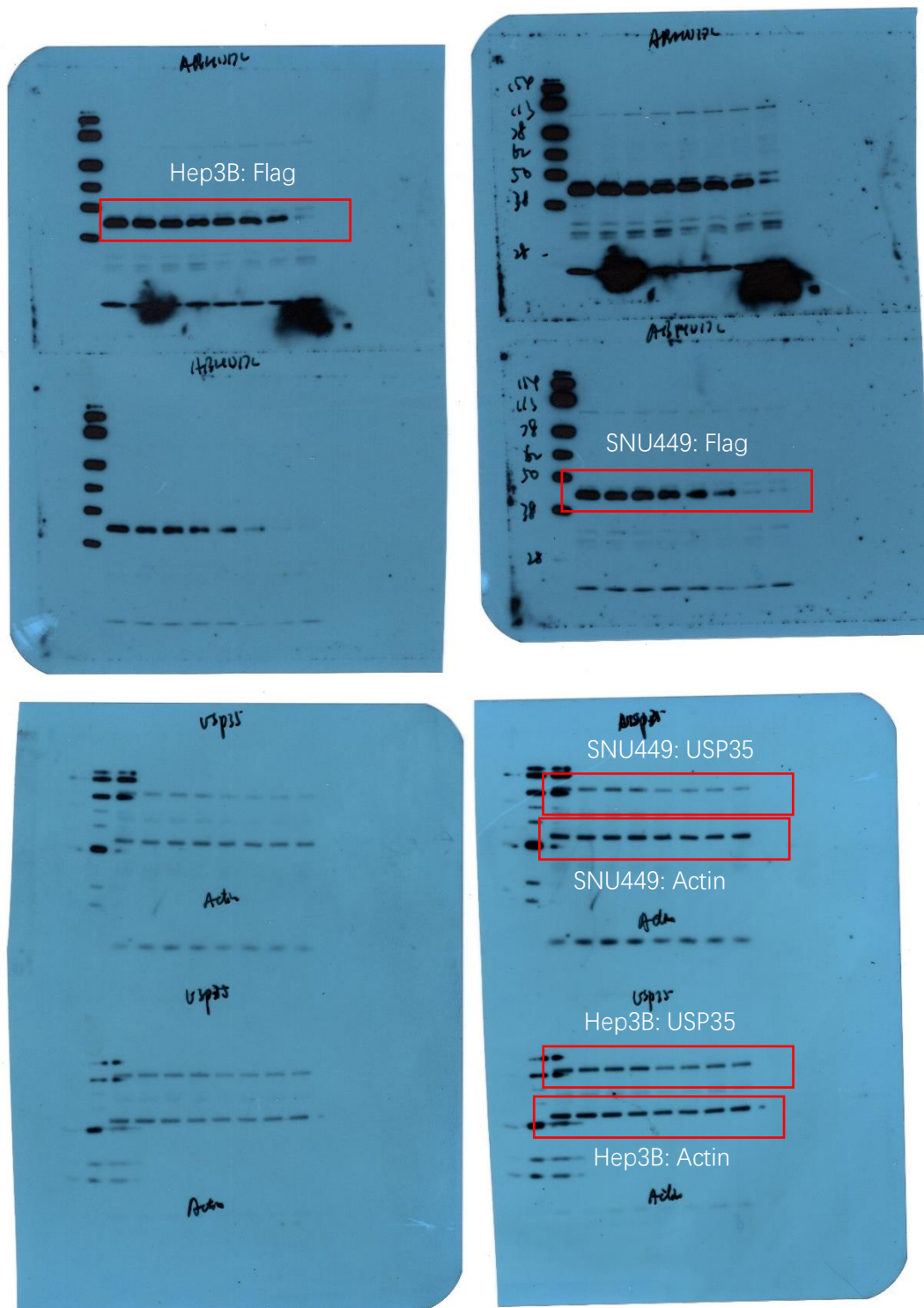

Figure 5A

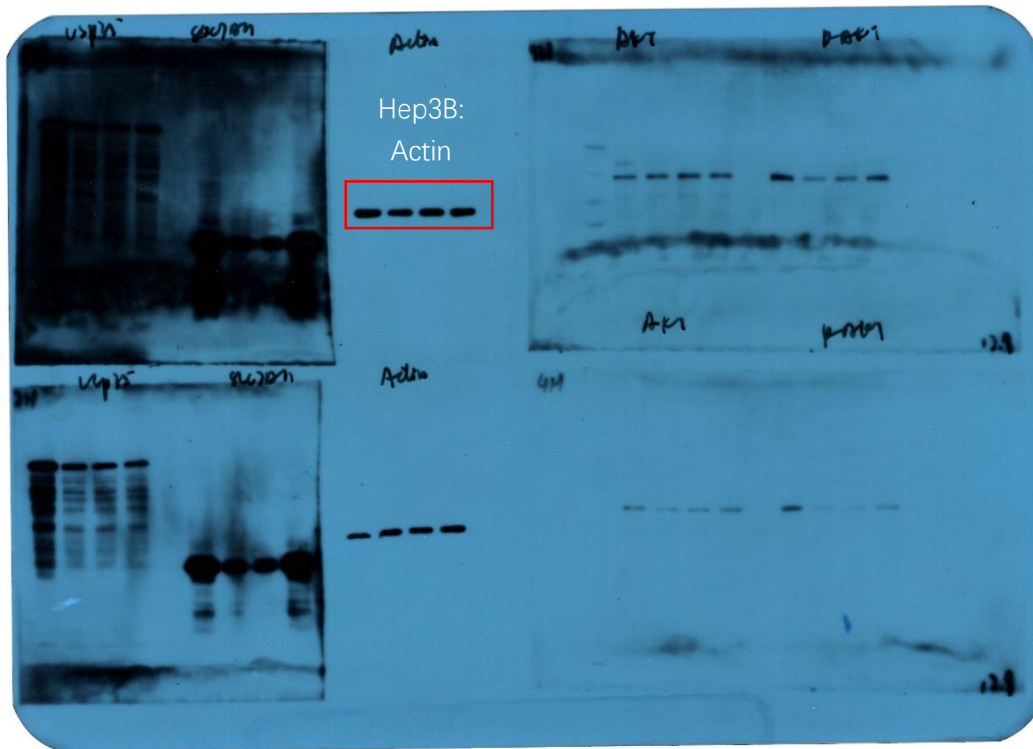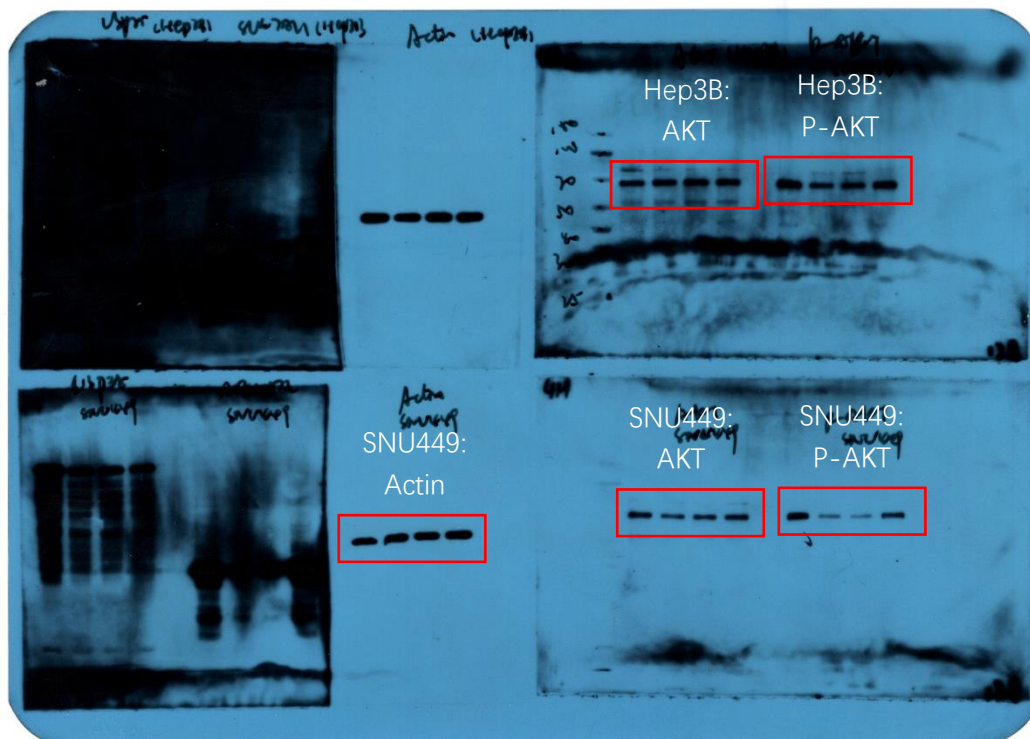

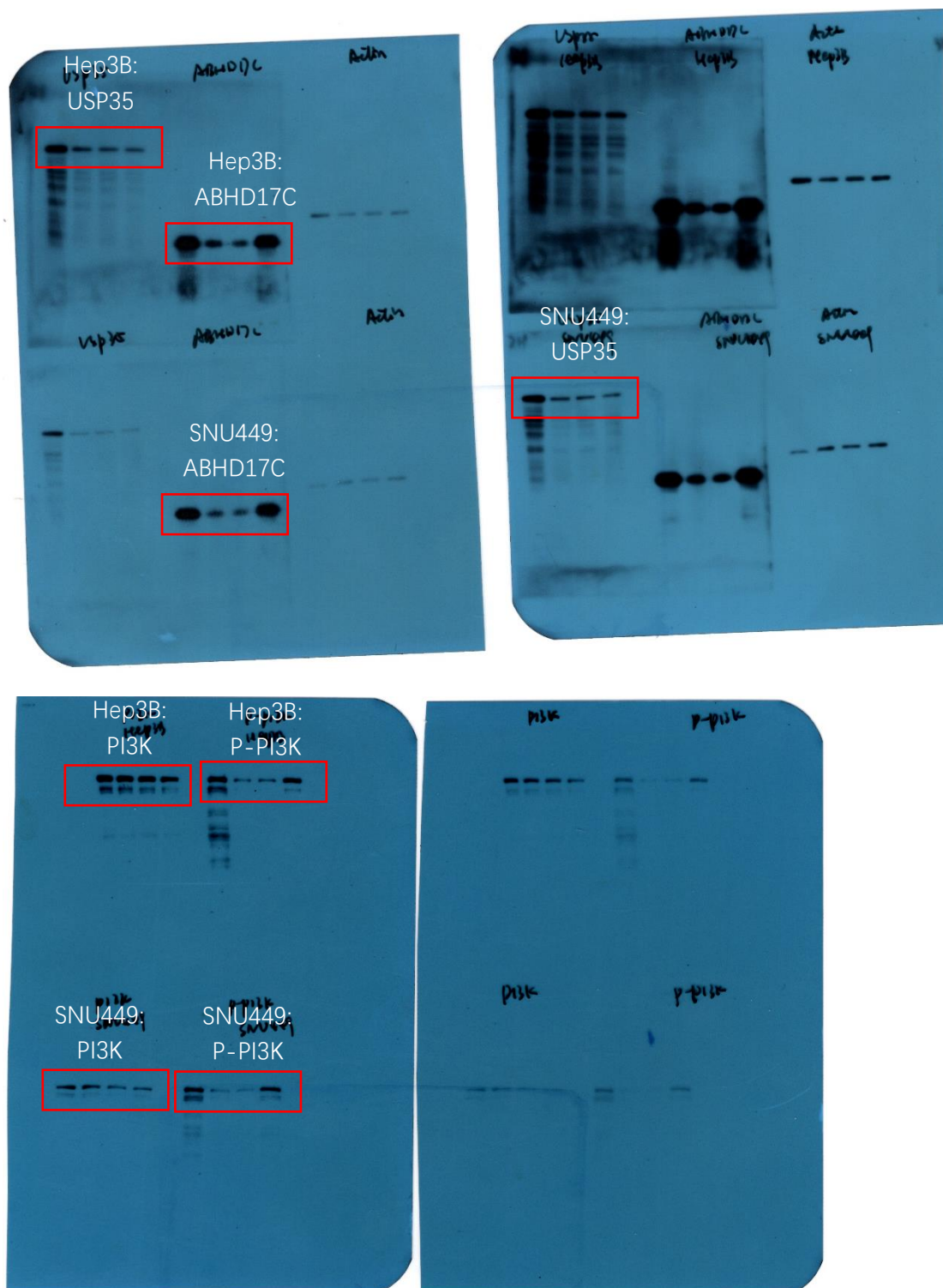

Figure 7H

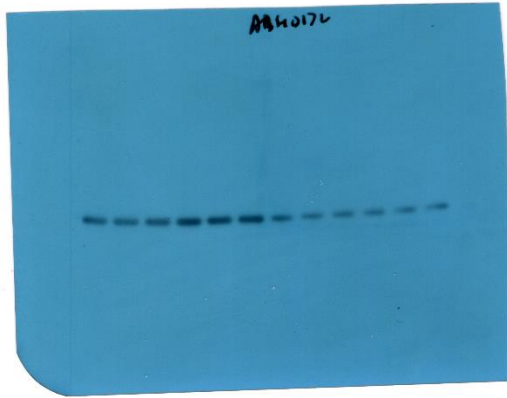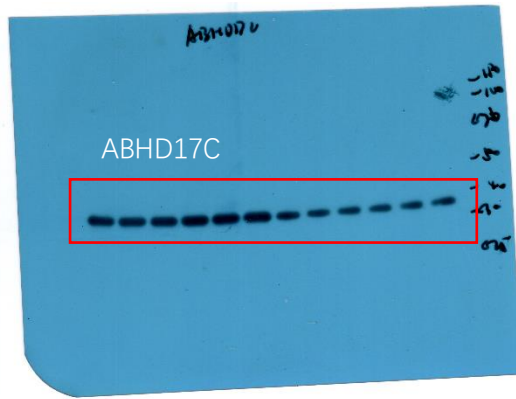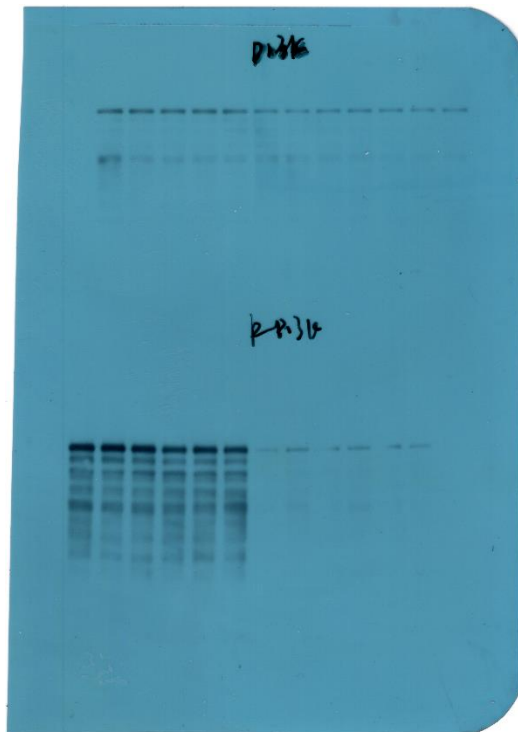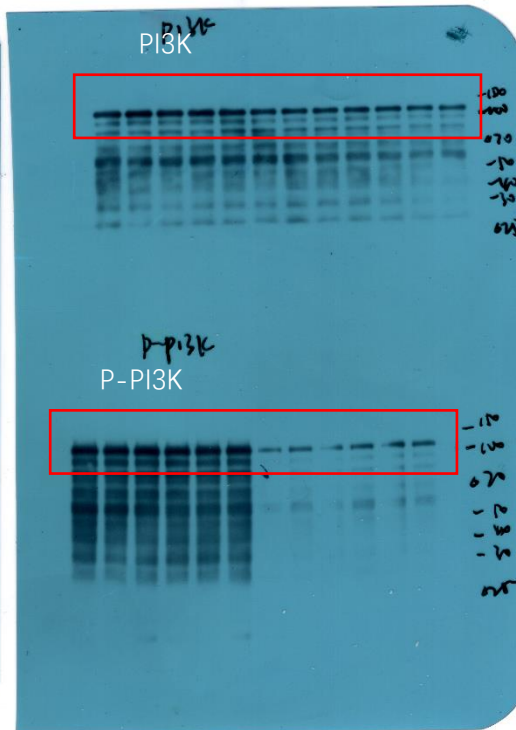

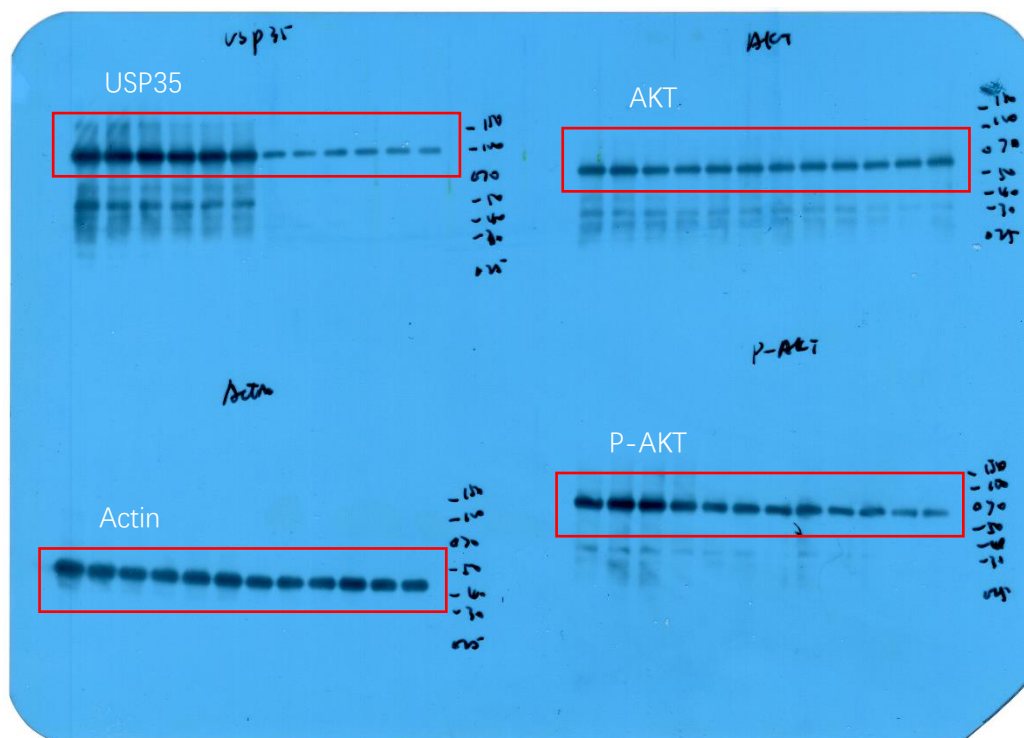

Figure S1

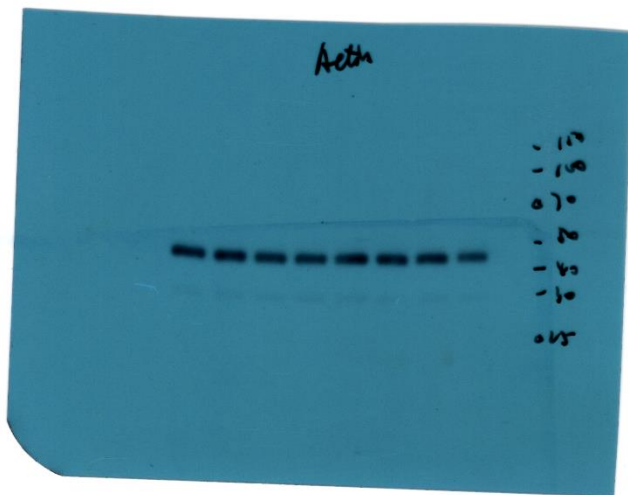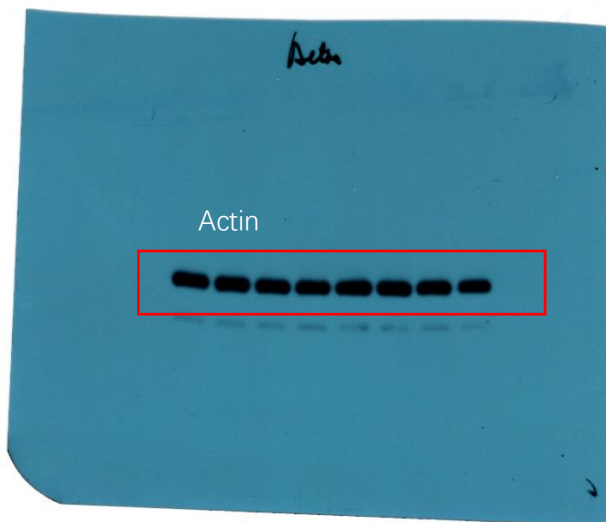

USP35

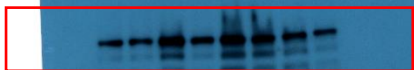

usp35

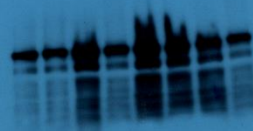

ABHD17C

ABHD17C

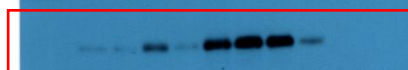

Supplement: Supplementary file 2 — Original Western Blot Data File [file 41420_2023_1714_MOESM2_ESM.pdf]
